# Changing the speed and order of attentional selection in visual search

**DOI:** 10.3758/s13423-024-02632-y

**Published:** 2025-02-18

**Authors:** Gregory J. Christie, Daniel Tay, John J. McDonald

**Affiliations:** Department of Psychology, Simon Fraser University, 8888 University Drive, Burnaby, BC V5A 1S6 Canada

**Keywords:** Visual search, Attention, ERPs and working memory/attention

## Abstract

Seminal event-related potential (ERP) studies of visual search reported that young adults serially inspect two singletons when searching for a target (serial search), but later results showed that the second singleton can be selected while the first singleton is still attended (partially parallel search). These contrasting results indicate that some yet-to-be identified factor can affect the speed of search. We hypothesized that single-target detection tasks promote serial inspection while dual-target comparison tasks promote parallel inspection. We recorded ERP activities associated with attentional selection (N2pc) and subsequent identification (SPCN) to track attentional processing of two singletons while healthy young adults participated in one of two detection tasks or a comparison task. One singleton was made to be more salient than the other to give it a “natural” selection advantage and thus promote some serial processing even in the comparison task. The timing of N2pc activities indicated that attention was deployed to the second singleton more quickly when participants compared the orientations of lines inside the two singletons than when they searched for one specific line that was more likely to be positioned inside one singleton or the other. Surprisingly, however, search was never fully serial, even in detection tasks that encouraged close inspection of individual items. Rather, in such detection tasks, items were selected serially but were processed for identification concurrently (as indexed by the SPCN). These findings are consistent with serial-parallel hybrid models of visual search.

**Supplementary Information:**

The online version contains supplementary material available at 10.3758/s13423-024-02632-y.

Humans regularly orient their attention to different locations of the visual field to find objects of interest. For example, a student might view numerous items on a desk in search of her orange phone. In this case, the individual might initially inspect a bright orange notepad because it resembles the phone. On another day, the student might purposely search for the notepad and the phone to take both items to campus. Would the individual find the phone faster in the latter situation, or would the speed of search be determined solely by the ease with which the two items can be distinguished from other items (i.e., salience)?

An event-related potential (ERP) component linked to attentional selection called the N2pc (Luck & Hillyard, 1994a, b) has been used to determine how quickly attention can be oriented from one potential target to another. In one series of studies, participants viewed displays containing two potential targets (colour singletons appearing near or far from fixation) and indicated whether a target-defining feature was present at one of the two locations (Woodman & Luck, 1999, 2003).^1^ On target-absent trials, it was presumed that participants would attend to both potential targets to determine if either one possessed the target-defining feature. On these trials, the potential target closest to fixation elicited an N2pc 200–375 ms after display onset, whereas the more distant one elicited an N2pc 375–550 ms after display onset. This pattern suggested that participants shifted attention serially from one potential target to another (Treisman & Gelade, 1980). However, a second series of studies demonstrated that attended items can elicit asynchronous-but-overlapping N2pc components rather than successive N2pc components (Grubert & Eimer, 2015). This latter pattern is consistent with a serial-parallel hybrid account of visual search, in which multiple items enter a central processing stage sequentially but remain in that central processor concurrently (Wolfe, 2003, 2007; Wolfe et al., 2010).

What aspect of the later study enabled participants to allocate attention to items more rapidly than in the former study? Grubert and Eimer (2015) speculated that differences in display complexity or target-identification difficulty may have been responsible for the contrasting results, but the stimuli and tasks used by the two research groups differed in numerous other ways. Here, we hypothesized that the type of tasks used in the two studies promoted the use of different strategies. As noted by Woodman and Luck (1999, 2003), their one-target detection tasks were purposely designed to impose a strictly serial inspection strategy. In contrast, Grubert and Eimer (2015; Eimer & Grubert, 2014) instructed participants to compare the identities (numeric values or alphanumeric categories) of two targets on every trial. We surmised that such a comparison task would promote parallel selection of two visual targets.

To evaluate this hypothesis, we presented participants with the same six-item search arrays in two detection tasks and one comparison task. Each display contained six shapes, including one colour singleton and one shape singleton. In the detection tasks, participants indicated the presence or absence of a target line (with prespecified orientation) that was more likely to appear within the colour singleton (C_75_ detection task) or the shape singleton (S_75_ detection task). In the comparison task, participants indicated whether lines contained within the two singletons had the same orientation or not. Display complexity was equated across all tasks, and differences in task difficulty were reduced by using the same type of target and nontarget stimuli (horizontal and vertical lines) in each task. Based on the contrasting results of prior studies, we predicted that the initial selection would be completely serial in the detection tasks but would be partially parallel in the comparison task. As in the previous studies, the N2pc was used to index the initial stage of attentional selection. In addition, we used the sustained posterior contralateral negativity (SPCN; Jolicœur et al., 2008; Mazza et al., 2007), which follows the N2pc, to index subsequent attentive processing associated with item identification (Fig. 1). This was done to determine whether participants fully identify the initially attended item before shifting attention to the next item, as would be predicted from a fully serial search model.

**Fig. 1:**
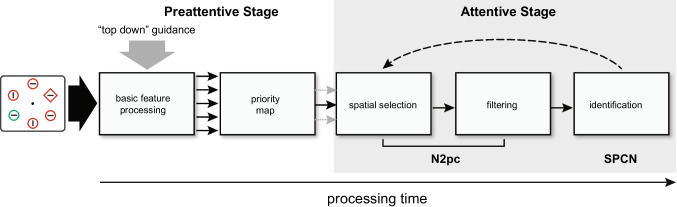
Hypothetical sequence of cognitive processes during the inspection of scenes containing multiple possible targets

A second goal of this study was to assess the roles of stimulus salience and top-down knowledge in the control of attention. According to salience-driven selection theory, participants should always attend to the most salient item before redirecting attention to a less-salient item (Theeuwes, 2010). Here, the item features were selected to ensure that the colour singleton was more salient than the shape singleton (Theeuwes, 1992), and so, according to salience-driven selection theory, participants should select the colour singleton first even when a to-be-detected target is more likely to be found in the shape singleton (S_75_ detection task). According to other theories, knowledge of the target’s likely location should enable selection of a less-salient item before a more salient item (e.g., Folk et al., 1992). We predicted that salience would “naturally” determine the order of selection in the comparison task, where both singletons are equally relevant, even though there was no strategic reason or explicit instruction to select one item before the other (see Christie et al., 2018), but that observers could override this tendency to orient attention to the less salient singleton in the S_75_ detection task.

## Methods

### Participants

The minimum sample size for each of the three experimental tasks was determined a priori to have sufficient power (1 − β = .80) to detect the presence of a small N2pc component by one-tailed, one-sample *t* tests (*d* = 0.58 in Tay et al., 2019). One-tailed tests were done because the N2pc is a negative voltage (i.e., its amplitude is expected to be more negative than zero microvolts). Using G*Power, we determined that a minimum sample size of 20 was needed, but we decided on a slightly larger sample size of 25 to be able to detect smaller N2pc components (*d* = 0.52). This sample size would be more than enough to detect differences between the onset latency of an early N2pc and a later N2pc (herein called N2pc onset asynchronies) in a two-tailed test, because such latency differences were expected to be large (*d* = 0.80; Grubert & Eimer, 2015; Woodman & Luck, 1999, 2003). Note that we first needed to statistically confirm the presence of each N2pc before testing for a latency difference, which is why we chose a priori to use a larger-than-minimum sample size.

In total, 98 participants were recruited and assigned randomly into one of three tasks: the C_75_ detection task, the S_75_ detection task, and the comparison task. All participants provided written consent, reported normal or corrected-to-normal vision, and were screened for colour blindness using Ishihara colour plates. Thirty-four observers participated in the C_75_ detection task. Data from nine participants were excluded due to excessive artifact (>25% of epochs contributing to ERPs), leaving 25 participants in the final sample for this task (mean age 20.7 years, 14 women, three left-handed). Thirty-one observers participated in the S_75_ detection task. Data from four participants were rejected due to excessive artifact, and one participant was excluded from the analysis due to poor task compliance. This left 26 participants in the final sample for this task (mean age 21.7 years, five men, five left-handed). Thirty-three observers participated in the comparison task. Data from four participants were excluded because accuracy was less than 70%, and data from four additional participants were excluded due to excessive artifact (more than 25% of trials rejected due to eye movements, blinking, or amplifier blocking). This left 26 participants in the final sample for this task (mean age 21.0 years, 12 women, two left-handed).

### Apparatus

All tasks were conducted in a dimly lit, acoustically and electrically shielded chamber illuminated by DC-powered LED lighting. Participants sat 57 cm from a computer monitor operating with an 85 Hz refresh. Stimulus presentation was controlled by Presentation (Neurobehavioural Systems Inc, Albany, CA) from a Windows-based computer. The EEG was recorded using custom software (Acquire) from a second Windows-based computer, using a 64-channel A-to-D board (PCI 6071e, National Instruments, Austin, TX) connected to a high (>1 Gohm) input impedance EEG amplifier system (SA Instruments, San Diego, CA).

### Stimuli and procedure

Prior to the commencement of the experiment, participants varied the brightness of a red box (0.9˚ × 1.0˚) so that it matched the brightness of a green box that was positioned immediately to the left. The brightness-matching process was repeated four times, and the average red value and fixed green value were used for red and green stimuli in the experiment proper (for methods, see Jannati et al., 2013).

Search displays contained six items (2.5° outer diameter, 0.2° line thickness) spaced equally around an imaginary circle (5.5° radius) centered on a fixation point (0.4° filled disc), with two items situated on the vertical meridian above and below the fixation point. Four of the items were unfilled circles rendered in the same colour (red or green, counterbalanced between participants). One item was a shape singleton (e.g., red diamond), and one other item was a colour singleton (e.g., green circle). Each item contained either a vertical line or horizontal line (1.3 × 0.1°). The orientations of lines inside the nonsingleton circles were set randomly and independently, whereas the orientations of lines inside the singletons depended on the task (see below). The background of the display was black (0.03 cd/m^2^), and the lines and fixation point were light gray (40 cd/m^2^). The brightness of the green items was fixed (26 cd/m^2^, u’ = .27, v’ = .61), and the brightness of the red items was matched to that of the green prior to the start of the experiment.

Each trial began with a fixation point displayed for 800–1,200 ms, followed by the search display. The search display would remain visible for four seconds or until participants pressed a button in response to the target. Participants were instructed to look at the central fixation cross and to maintain eye fixation throughout the experiment. Four display configurations were equally likely and randomly presented within each block of 32 trials: lateral-colour-singleton/midline-shape-singleton, lateral-shape-singleton/midline-colour-singleton, lateral singletons on same side, and lateral singletons on opposite sides. Participants completed 32 blocks for a total of 1,024 trials (256 trials of each display configuration), with participant-controlled rest periods between each block.

For the C_75_ and S_75_ detection tasks, participants were required to press one of two buttons depending on the presence or absence of a single target line with a prespecified orientation (vertical or horizontal; counterbalanced between participants). The target line was present or absent on an equal proportion of trials. In the C_75_ task, the target was located within the colour singleton on 75% of the target-present trials and within the shape singleton on the remaining 25% of target-present trials. In the S_75_ task, the target was located within the shape singleton on 75% of target-present trials and within the colour singleton on the remaining 25% of target-present trials. Participants were informed of these probabilities at the outset of the experiment but were not instructed to search for the target in any particular order.

For the comparison task, the stimuli and procedures were identical to the detection tasks except for the following changes. Participants were instructed to press one of two buttons as quickly as possible depending on whether the lines contained within the two singletons had the same orientation or different orientations. Thus, unlike the detection tasks, there was no predesignated target line orientation and there were no target-absent trials. Participants were given no other instructions on how best to perform the comparison judgment and were not instructed to attend to the two singletons in any particular order.

### Behavioural analysis

Response times (RTs) from the two detection tasks were submitted to an analysis of variance (ANOVA), with a between-subjects factor for task (C_75_, S_75_) and a within-subjects factor for target condition (more likely location; less likely location; absent). Planned, pairwise comparisons were made using the Bonferroni correction to control for the familywise error rate (all reported *p* values were adjusted). RTs from the comparison task were assessed by an ANOVA with one within-subject factor for display configuration (same side, opposite sides, lateral-colour/midline-shape, lateral-shape/midline-colour). RTs from the comparison task were analyzed separately because we had no a priori predictions about how performance in that task would compare with performance in the two detection tasks. Error rates from the two detection tasks and from the comparison task were assessed following the same ANOVA strategies used to assess RTs.

### Electrophysiological recording and analysis

The electroencephalogram (EEG) was recorded from Ag/AgCl electrodes mounted in an elastic cap (Sands Research, Inc.) from 24 scalp locations: FP1, FPz, FP2, F7, F3, Fz, F4, F8, T7, C3, Cz, C4, T8, P7, P3, Pz, P4, P8, PO7, POz, PO8, O1, Oz, and O2. An additional electrode was attached to the left mastoid (M1). These electrodes were referenced to an electrode on the right mastoid during recording and were re-referenced offline to the average of the two mastoids. A separate bipolar channel was used to record the horizontal electrooculogram (HEOG) from Ag/AgCl electrodes positioned 1 cm lateral to the outer canthus of each eye. EEG and HEOG channels were amplified with a gain of 20,000 within a pass-band of 0.01–100 Hz (two-pole Butterworth filters) and were digitized at 500 Hz. A semiautomatic procedure was performed to remove epochs of EEG that were contaminated by horizontal eye movements, blinks (and vertical eye movements), and amplifier blocking. Artifact-free data were then used to create averaged ERP waveforms, which were digitally low-pass filtered (half-amplitude cutoff at 30 Hz) to remove high-frequency activity. A 200-ms prestimulus interval (−200–0 ms) was used for baseline correction. The averaged event-related HEOG did not exceed 3 μV for any individual participant that remained in the samples, indicating that gaze remained within 0.5° of the fixation point for most trials (McDonald & Ward, 1999).

ERPs time-locked to the onset of the search displays were computed separately for the two display configurations that enabled us to ascribe any observable N2pc to one singleton (lateral-colour/midline-shape) or the other (lateral-shape/midline-colour; for methods, see Hickey et al., 2009; Woodman & Luck, 2003). Only correct-response trials were used to compute ERPs. For each participant, the ERP waveforms were collapsed across left and right visual hemifields and left and right electrode sites to create waveforms recorded contralateral and ipsilateral to the lateral singleton. Lateralized ERP difference waveforms were then derived for each of the two display configurations of interest by subtracting the ipsilateral waveform from the corresponding contralateral waveform using lateral occipital electrode sites PO7 and PO8. These electrodes were selected a priori on the basis of numerous N2pc studies from our lab and elsewhere (e.g., Gaspar & McDonald, 2014; Tay et al., 2019). Negative voltages were plotted upward such that the N2pc and SPCN would appear as upward deflections in these difference waveforms.

All N2pc and SPCN measurements were taken from the contralateral-ipsilateral difference waves at PO7/PO8. The timing of the N2pc was expected to vary across display configurations and tasks, and so mean amplitudes of the N2pc peaks were measured after finding the onset latency of each individual N2pc. The onset latency was determined in a two-step process. First, the first local negative peak within a 100–425-ms interval was found using three sample points around the local peak to prevent selection of the end of the measurement window. This interval was wide enough to find the two successive N2pc peaks in Woodman and Luck’s (2003) study. We picked the first local peak rather than the largest (i.e., most negative) local peak because the N2pc is usually followed by the SPCN at the same lateral occipital electrodes.^2^ The onset latency of the N2pc was then defined as the time at which the amplitude reached 33% of the local peak amplitude. To characterize the magnitude of each N2pc, we computed the mean amplitude in a 75-ms window starting at the peak’s onset latency. When possible, offset latencies of the N2pc were measured as the times at which the postpeak waveform first reached 33% of its local peak amplitude (note: this was not possible when the N2pc merged with the later SPCN). All latency measures were taken from jackknife (*N* − 1) subaverages rather than individual-participant difference waves to avoid difficulties running statistical tests when a peak cannot be found in an individual data file (due to noise or absence of component; Miller et al., 1998). Differences in N2pc latencies were tested statistically using conventional adjustments for the jackknife approach (Miller et al., 1998; Smulders, 2010; Ulrich & Miller, 2001). The presence of N2pc was evaluated statistically using a one-sample *t* test versus 0 µV. Uncorrected degrees of freedom are reported for simplicity. Finally, the amplitude of the SPCN was measured in a relatively late 500–600-ms interval to avoid conflating the SPCN with late-onset N2pc waves (Jolicœur et al., 2008, used 450–600 ms; Mazza et al., 2007, used 350–600 ms). One-sample *t* tests for N2pc and SPCN amplitudes were one-tailed tests because the components were expected to be negative (i.e., less than 0 μV; Tay et al., 2022a, b; Tay et al., 2022a, b).

## Results

### Behaviour

**Fig. 2:**
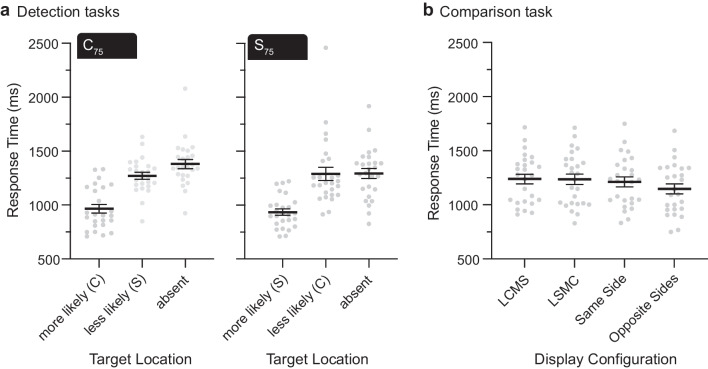
Response times (RTs) in ms. Gray dots represent mean RTs for individual participants, solid horizontal lines represent grand-averaged (across participants) RTs, and error bars represent *SEM*s. **a** Results from C_75_ and S_75_ detection tasks. When present, targets were more likely or less likely to be located within the colour singleton (C) or the shape singleton (S). **b** Results from the comparison task. LCMS = lateral colour singleton, midline shape singleton; LSMC = lateral shape singleton, midline colour singleton

Figure 2 displays RTs for each task, after rejecting trials contaminated by artifact (21.8%, 16.8%, and 14.4% for the C_75_ detection task, and S_75_ detection task, and comparison task, respectively) and removing outliers (±2 *SD*s from their respective means). Grand averaged RTs and errors rates are provided in Supplementary Table 1.

#### **Detection tasks**

RTs differed across target conditions, *F*(2,98) = 73.1, *p* < .001, η_p_^2^ = 0.38. Neither the main effect of task, *F*(1,49) = 0.51, *p* = .477, nor the Task × Target Condition interaction, *F*(2,98) = 1.18, *p* = .307, was significant. All pairwise comparisons were significant, indicating that RTs were shortest when the target appeared within the more-likely singleton (i.e., colour singleton in the C_75_ detection task; shape singleton in the S_75_ detection task), intermediate when the target appeared within the less-likely singleton, and longest when the target was absent, *t* values ≥ 9.56, *p* values < .001, *d*s ≥ 1.50. Differences in error rates gave rise to a main effect of target condition, *F*(2,98) = 32.5, *p* < .001, η_p_^2^ = 0.26, and a Task × Target Condition interaction, *F*(2,98) = 5.41, *p* = .023, η_p_^2^ = 0.04 (with a larger difference in the S_75_ detection task than in the C_75_ detection task). The error rates did not differ across tasks, *F*(1,49) = 1.75, *p* = .192. Pairwise comparisons revealed that participants made more errors when the target appeared within the less-likely singleton than when the target appeared in the more-likely singleton or was absent, *t* values ≥ 6.83, *p* values < .001, *d*s ≥ 1.25. These behavioural results indicate that participants first searched the singleton that was more likely to contain the target line, regardless of the singleton’s salience. Additionally, participants were most likely to miss the target when it was expected to appear within the (less salient) shape singleton but appeared within the (more salient) colour singleton.

#### **Comparison task**

RTs varied across the four display configurations, *F*(3,75) = 18.89, *p* < .001, η_p_^2^ = 0.43. Pairwise comparisons revealed that the mean RT was significantly shorter for the opposite-sides configuration than for each of the other configurations, adjusted *p* values < .001. No other pairwise difference was significant. These results indicate that the orientation comparison was easiest when the two singletons were in different hemifields. Error rates also varied across display configurations, *F*(3,75) = 5.76, *p* = .002, η_p_^2^ = 0.19, with the lowest rate in the opposite-sides configuration. Pairwise comparisons revealed fewer errors in the opposite-sides configuration than in the lateral-colour/midline-shape configuration and the lateral-shape/midline-colour configuration.

### ERPs

**Fig. 3:**
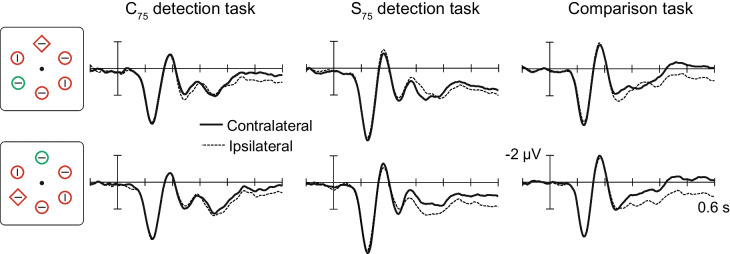
Event-related potentials elicited by search displays of interest for the C_75_ detection task, S_75_ detection task, and comparison task. Waveforms were recorded from occipital electrodes (PO7/PO8) positioned ipsilateral and contralateral to the lateral singleton in each display. For the C_75_ and S_75_ detection tasks, ERP waveforms were obtained from trials on which the target line was absent in either of the singletons

**Fig. 4:**
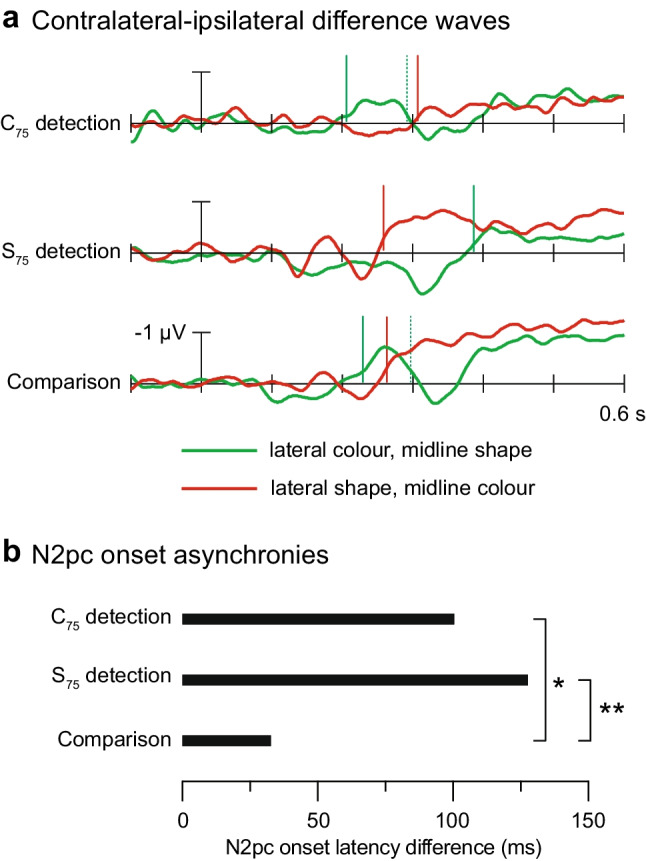
N2pc and SPCN results. **a** Contralateral-minus-ipsilateral difference waveforms corresponding to the event-related potential waveforms displayed in Fig. 3. Solid vertical lines denote N2pc onset latencies, and dashed vertical lines denote N2pc offset latencies. **b** N2pc onset asynchronies across tasks. Each bar represents the difference in ms between the onset of the second N2pc (e.g., elicited by shape singleton in C_75_ detection task) and the onset of the first N2pc (e.g., elicited by colour singleton in C_75_ detection task). *p < .05; **p < .001

Figure 3 displays grand-averaged occipital ERP waveforms elicited by search displays containing one lateral singleton (and one midline singleton). The contralateral and ipsilateral waveforms largely overlapped in the time range of the P1 (120 ms) and N1 (190 ms) peaks. At some point after the N1, the contralateral waveforms became more negative than the ipsilateral waveforms. Such differences can be ascribed to the lateral singleton because items appearing on the vertical meridian do not trigger lateralized ERP components associated with attention or suppression (Hickey et al., 2009; Woodman & Luck, 1999, 2003). Contralateral-ipsilateral difference waves were computed to examine the timing of these differences (Fig. 4a).

#### N2pc: C_75_ detection task

The lateral colour/midline-shape display elicited an N2pc in the conventional time range on target-absent trials. The onset and offset latencies of this colour-singleton N2pc were 206 ms and 292 ms, respectively. The voltage was significantly more negative than zero within the 75-ms measurement window, *t*(24) = 2.40, *p *= .012, *d* = 0.48, confirming the presence of the colour-singleton N2pc. The lateral-shape/midline-colour display also elicited a contralateral negativity in the C_75_ detection task, *t*(24) = 1.79, *p* = .043, *d* = 0.36, but its onset latency was significantly longer (307 ms) than that of the colour-singleton N2pc, *t*(24) = 3.19, *p* = .004, *d* = 0.64. In fact, the onset of the shape singleton N2pc trailed the offset of the colour-singleton N2pc by 15 ms, although statistically no difference was found, *t*(24) = 1.28, *p* = .212. These results indicate that the colour singleton was selected first and that the shape singleton was selected only after selection of the colour singleton was complete.

#### N2pc: S_75_ detection task

Each of the two singletons elicited an N2pc, colour singleton: *t*(25) = 2.65, *p* = .007, *d* = 0.52; shape singleton: *t*(25) = 3.90, *p* < .001, *d* = 0.77. Critically, however, the order of these two N2pc components was reversed relative to the order obtained in the C_75_ detection experiment. The shape-singleton N2pc emerged 128 ms before the colour-singleton N2pc (259 ms vs. 387 ms, respectively), *t*(25) = 13.69, *p* < .001, *d* = 2.68. The differences in onset latencies across detection tasks were also statistically significant for the lateral colour singleton (181 ms later in the S_75_ detection task than in the C_75_ detection task), *t*(49) = 5.74, *p* < .007, *d* = 1.61, and the lateral shape singleton (48 ms earlier in the S_75_ detection task than in the C_75_ detection task), *t*(49) = 5.17, *p* < .001, *d* = 1.45. These results indicate that the order of selection was determined by the target’s most likely location rather than stimulus salience.

#### N2pc: Comparison task

The results from the C_75_ and S_75_ detection tasks were consistent with Woodman and Luck’s (1999, 2003) findings. In those tasks, potential targets elicited sequential N2pc components, with N2pc onsets separated by 100–128 ms (Fig. 4b). The results of our comparison task were different, even though participants searched the exact same two-singleton displays. As before, each singleton was found to elicit an N2pc, colour singleton: *t*(25) = 2.61, *p* = .015, *d* = −0.51; shape singleton: *t*(25) = 3.74, *p* < .001, *d* = −0.73. Participants were not instructed to search in any particular order, but the onset latency of the colour-singleton N2pc was significantly shorter than that of the shape-singleton N2pc (230 ms vs. 263 ms, respectively), *t*(25) = 2.95, *p* = .007, *d* = 0.58. Critically, this 33-ms N2pc onset asynchrony was significantly smaller than the N2pc onset asynchronies obtained in the C_75_ detection task (101 ms), *t*(49) = 2.06, *p* = .045, *d* = 0.58, and in the S_75_ detection task (128 ms), *t*(50) = 6.54, *p* < .001, *d* = 1.82. Moreover, unlike in the detection tasks, the second N2pc (here elicited by the shape singleton) began before the first N2pc ended (263 onset vs. 297 ms offset), *t*(25) = 3.47, *p* = .002, *d* = 0.68. This pattern is consistent with the asynchronous-but-overlapping N2pc waves observed in Grubert and Eimer’s (2015) comparison task.

#### SPCN: Across all tasks

The SPCN sometimes appeared as a second, separate negativity in the contralateral-ipsilateral difference waves. For example, in the C_75_ detection task, the lateral-colour/midline-shape display elicited an early N2pc and a separate SPCN starting at approximately 400 ms. At other times, a late N2pc merged with the SPCN. Therefore, we measured SPCN in a late interval (500–600 ms) to avoid conflation with N2pc. Lateral-colour/midline-shape and lateral-shape/midline-colour displays were found to elicit the SPCN in the C_75_ detection task, *t*s(24) ≥ 2.90, *p* values ≤ .004, *d* ≥ 0.58, the S_75_ detection task, *t*s(25) ≥ 2.30, *p* values ≤ .015, *d*s ≥ 0.45, and the comparison task, *t*s(25) ≥ 4.07, *p* values < .001, *d*s ≥ 0.80. That is, the two singletons elicited concurrent SPCNs in each task, which indicates that lines located within the two singletons were processed for identification concurrently, even in the detection tasks.

## Discussion

Our results confirm that task requirements strongly influence whether attention can be deployed to the second of two singletons before selection of the first singleton is complete. When the task was to detect a specific line that was more likely to appear within one of the singletons, the two items elicited sequential N2pc components, with over 100 ms between N2pc onsets (as in Woodman & Luck, 1999, 2003). This pattern indicates that the second singleton was selected for identification only *after* selection of the first singleton was complete. In contrast, when the task was to compare line orientations within the two singletons, those items elicited asynchronous-but-overlapping N2pc components (as in Eimer & Grubert, 2014; Grubert & Eimer, 2015). This finding indicates that the second singleton was selected for identification *before* selection of the first singleton was complete. The N2pc onset asynchrony was reduced from over 100 ms in the detection tasks to just 33 ms in the comparison task. These findings demonstrate that the speed of attentional selection depends on task demands. More specifically, the results support our hypothesis that two-item comparison tasks promote fast, parallel selection.

### Reconsidering ERP evidence for serial search

The nonoverlapping N2pc components appear to suggest that the potential targets were inspected serially in the two detection tasks (also see Woodman & Luck, 1999, 2003). By this serial-search account, participants in the C_75_ detection task first identified the nontarget line within the colour singleton before selecting and identifying the line within the shape singleton. For this serial-search interpretation to be viable, however, one must assume that the N2pc reflects the entire duration of attentive processing. The N2pc was preceded by no other lateralized selection negativity (e.g., N1pc) in the current study, so it is reasonable to assume the onset of N2pc accurately reflected the start of attentional processing here (Jannati et al., 2013; Töllner et al., 2012).^3^ The assumption that the N2pc offset marked the cessation of attentive processing is less tenable, however, because the N2pc is hypothesized to reflect an early stage of attentional selection or engagement, not necessarily the entire duration of attentive processing (Eimer & Kiss, 2010; Jannati et al., 2013; Luck & Hillyard, 1994a, b; Zivony et al., 2018). The N2pc is followed by the SPCN in tasks that require discrimination of the target’s identity or features (Mazza et al., 2007), and the amplitude of the SPCN increases when the difficulty of target identification increases (Töllner et al., 2013). These findings suggest that the SPCN reflects perceptual analysis of the attended item after initial selection (i.e., further attentive processing). As depicted in Fig. 1, we consider the combined duration of the N2pc and SPCN to more accurately reflect the duration of attentive processing (i.e., *attentional dwell time*; Duncan et al., 1994). Critically, regardless of the order and timing of the N2pc components, the two singletons elicited concurrent SPCN components 500–600 ms after display onset. A similar pattern of results can be seen in Woodman and Luck’s (2003, Experiment 2) ERP waveforms, although there was no measurement of SPCN in that study.

Together, our ERP findings are more consistent with hybrid search (Wolfe, 2003) than with purely serial search, even when the task encouraged serial inspection of potential targets. According to this hybrid model, "identification of one object need not end before the process begins for the next item. Attention can be deployed to the next item while the prior item is still in the process of being identified. At that point, two items would be in the identification process at the same time” (Wolfe et al., 2010, p.). By this view, the concurrently elicited SPCN waves in our two detection tasks indicate that the two potential targets were being identified simultaneously (in the 500–600-ms time range) despite being selected sequentially.

### On the role of salience in determining initial deployment of attention

According to *salience-driven selection theory*, salient items capture attention automatically whenever participants are set to search for a sufficiently salient target (Theeuwes, 1992, 2010). By this account, one might have predicted the more salient colour singleton to elicit the earliest N2pc in all tasks, regardless of the target’s likelihood of being located within that singleton. However, the less salient shape singleton was found to elicit the earliest N2pc in the S_75_ detection task. Moreover, the onset latency of the colour-singleton N2pc varied by 181 ms across the two detection tasks, even though identical displays were used. These results indicate that participants can overcome salience-driven selection to search for less salient objects of interest.

## Supplementary Information

Below is the link to the electronic supplementary material.

Supplementary file1 (DOCX 15 KB)

## Data Availability

The EEG files are available upon request to the corresponding author. Processed data, including RTs, ERP component latencies, and ERP component amplitudes are available online (https://osf.io/qn63m).

## Footnotes

1 In another experiment, Woodman and Luck (1999) used displays containing four different-coloured singletons and biased search toward one of the colour singletons by making that item the target on 75% of target-present trials.

2 The initial phase of the sustained contralateral negativity was assumed to be an N2pc whenever a preceding N2pc peak was not evident (see also Grubert & Eimer, 2015; Woodman & Luck, 2003).

3 The shape singleton appeared to elicit a contralateral positivity prior to the N2pc in some of the tasks. These positivities might reflect a component called the distractor positivity (P_D_), which is hypothesized to reflect suppression (Gaspar & McDonald, 2014; Hickey et al., 2009). We refrained from testing or interpreting these positivities because we had no a priori hypotheses about the P_D_. These positivities did not overlap with our N2pc measurement windows, and so there was no need to alter our a priori plan to use one-tailed tests to detect the presence of N2pc.

## Code availability

Computer-controlled experiments written and run in Presentation (Neurobehavioral Systems, Inc) are available upon request to the corresponding author. All analysis scripts are available upon request to the corresponding author.

## Declarations

### Author note

This study was supported by grants from the Natural Sciences and Research Council of Canada, Canadian Foundation for Innovation, and the Canada Research Chairs program.
