# Supplementary material for: Changing the speed and order of attentional selection in visual search
Source: Psychon Bull Rev. 2025 Feb 18;32(4):1740–9. doi: 10.3758/s13423-024-02632-y (PMC12325472; doi:10.3758/s13423-024-02632-y)
Supplement: Supplementary file 1 — Supplementary file1 (DOCX 15 KB) [file 13423_2024_2632_MOESM1_ESM.docx]

Supplementary Table 1

*Median Correct Response Times* (*RTs) and Error Rates by Experimental Task*

| Task / Display Configuration | | RT (SEM)  ms | Error rate (SEM)  % |
| --- | --- | --- | --- |
| C_75_ task | |  |  |
|  | Target in colour singleton (more-likely) | 996 (39.5) | 4.3 (0.57) |
|  | Target in shape singleton (less-likely) | 1271 (32.5) | 10.3 (1.23) |
|  | Target absent | 1380 (41.9) | 3.4 (0.55) |
| S_75_ task | |  |  |
|  | Target in shape singleton (more-likely) | 935 (28.9) | 3.0 (0.53) |
|  | Target in colour singleton (less-likely) | 1289 (62.2) | 18.3 (3.74) |
|  | Target Absent | 1292 (46.6) | 3.0 (0.54) |
| Comparison Task | |  |  |
|  | Lateral colour singleton, midline shape | 1239 (44.3) | 7.6 (0.93) |
|  | Lateral shape singleton, midline colour | 1236 (47.7) | 7.2 (1.04) |
|  | Singletons on same sides | 1212 (45.7) | 6.9 (0.85) |
|  | Singletons on opposite side | 1148 (46.5) | 5.3 (0.80) |
